# Understanding COVID-19 Vaccine Hesitancy Through an Organizational Behaviour Lens

**DOI:** 10.7759/cureus.29459

**Published:** 2022-09-22

**Authors:** Yasir H Khan, Drew MacNeil, Jessica Bigelow, Melissa-Zoraya Corvalan Cifuentes, Christine Rottar

**Affiliations:** 1 Faculty of Health Sciences - Healthcare Quality Program, Queen's University, Kingston, CAN; 2 Department of Family and Community Medicine, University of Toronto, Toronto, CAN

**Keywords:** vaccine hesitancy, organizational behaviour, cognitive bias, groupthink, individualism, motivation, perception, covid-19 retro

## Abstract

Vaccine hesitancy during the COVID-19 pandemic has been a worldwide public health challenge. Organizational behaviour, the study of people’s behaviours in organizational settings, can be used to identify the behavioural drivers contributing to vaccine hesitancy and to develop targeted strategies to combat those drivers and improve vaccine uptake. Some common behaviours driving vaccine hesitancy arise from individualism, motivation, attitude, perception, groupthink, heuristics and cognitive bias. Organizational behaviour strategies to combat vaccine hesitancy include fostering a collectivist attitude, overcoming personal barriers to communication such as individual beliefs and values, utilizing motivation theories to target the individualistic mindset, and overcoming attitudes and perceptions by addressing heuristics and cognitive biases.

## Editorial

The COVID-19 pandemic has brought vaccine hesitancy back to the forefront as a worldwide public health challenge. In order to improve vaccine uptake and limit the spread of communicable diseases, we must explore the behavioural drivers that cause people to defer receiving vaccines, including the COVID-19 vaccine. Understanding these drivers from an organizational behaviour perspective can ultimately be used to reduce vaccine hesitancy and improve uptake. Organizational behaviour consists of the study of individual and group behaviours in organizational settings; it is utilised to explain why people behave the way they do in these settings [1]. Organizational behaviour is also utilized to predict human behaviour and to give managers tools and strategies to help manage the behaviour of people in these settings. In the context of the COVID-19 pandemic, these principles can be considered from an overall public health perspective as well as a health management perspective in settings where institutions implement immunization policies that need to be enforced by staff. The recommendations provided here to address vaccine hesitancy can help provide healthcare and organizational leaders with a framework to address this problem.

Behavioural drivers of vaccine hesitancy

Some of the common behaviours driving vaccine hesitancy arise from individualism, motivation, attitude, perception, groupthink, heuristics and cognitive bias.

Individualism refers to a loose social framework in which individuals focus on the well-being of themselves and their immediate family; in contrast, collectivism refers to tighter social frameworks that value group interest and what is best for the larger community rather than self-interest [1]. To see the impacts of individualism on vaccine hesitancy, we can analyze patterns of vaccination as well as COVID-19 outbreaks throughout the globe. One study looked at many health beliefs using an online questionnaire and found that people who held strong collectivist and altruistic ideas, in general, were also more likely to have higher vaccination intention [2].

Another study examined data from 69 countries in an attempt to establish links between societal values such as individualism or collectivism and the impact of COVID-19 [3]. Researchers used the Hofstede individualism score in comparison to COVID-19 mortality data. This study found that more individualistic countries, such as the United States, Great Britain, Italy, and Belgium had higher rates of COVID-19 cases as well as higher mortality. In contrast, countries that were more collectivist, such as Colombia and Korea, had amongst the lowest COVID-19 mortality rates early in the pandemic. Furthermore, when comparing a moderately individualistic country (Israel) and a highly individualistic country (USA), authors found that participants from the more individualistic country were less likely to indicate adherence to prevention measures such as masking and social distancing. It is worth noting that in this study there were no countries identified with a highly collectivist orientation that also had high COVID-19 mortality rates early in the pandemic. However, the study did identify some highly individualistic countries such as Canada, Australia and New Zealand that also had very low covid mortality rates early in the pandemic. These country-specific differences could have been the result of public health measures and policies that were implemented to limit the spread of COVID-19.

From a motivation perspective, as Alderfer and Maslow have established, basic needs of the individual, such as safety and security, must often be fulfilled first [4,5]. Once these needs are met, individuals may progress to development of higher order needs which may include a level of growth and belongingness leading to motivate more altruistic behaviour. For example, once basic physiological needs such as shelter, food and water have been met, an individual can move to safety needs such as psychological and physical security. Once those needs are met, individuals can then progress to needs of relatedness such as love and social belonging. However, for individualistic persons, they may feel that public health and vaccine mandates are challenging even their basic security needs by infringing upon their autonomy and perception of personal freedom. Vroom’s expectancy theory posits that motivation is a result of an individual’s belief of the connection between their effort and performance, and how an end result can be impacted by that performance [6]. In this context, individuals may be less motivated to follow public health guidance and vaccine recommendations if they are not convinced that those recommendations will improve their health outcomes, or feel that those recommendations may infringe upon other areas of their life.

Motivation as a behavioural driver also comes into play when addressing complacency, as it is strongly associated with lower vaccine uptake [7]. Complacency in this context includes the perception of low personal risk or low disease severity. This phenomenon of complacency has been reported more frequently in certain populations such as younger people and those of low socioeconomic status [7]. When individuals are indifferent toward receiving the COVID-19 vaccine based on the expected outcome and when motivation is lacking due to a perception of low disease risk or severity, this can further contribute to vaccine hesitancy.

The attitude of distrust in vaccination is another important behavioural driver behind vaccine hesitancy related to the COVID-19 vaccine. An individual’s attitude is their mind-set or tendency to act in a fairly consistent way toward an object or situation, due to their beliefs, values, experiences and temperament [1]. Beliefs about the risks and side effects of receiving the COVID-19 vaccine, along with feelings, knowledge, and awareness about health and disease prevention, are all factors that can contribute to an attitude of distrust and vaccine hesitancy [8].

Perceptions regarding concerns of vaccine safety, efficacy and long-term side effects are amongst the strongest predictors of COVID-19 vaccine hesitancy [7]. Perceptions are formed unconsciously; they arise from a process by which individuals interpret and understand a given situation based on previous experiences, often with incomplete information which can lead to inaccuracy [1]. People tend to form judgements that are convenient or acceptable to them when making decisions. This can occur when individuals selectively amplify or accept the aspects that support their position and ignore or downplay information that does not, which increases certainty in decision-making, although it may not be based on reality. Perceptions around concerns of vaccine safety and efficacy may be formed by a multitude of factors including experiences with vaccination and the healthcare system, cultural or religious background, political influencers, traditional and social media, and individual personality traits [7,8].

Just as individuals are prone to cognitive biases when making personal decisions, groups are also prone to a different set of biases including groupthink, group polarization, and escalation of commitment [9]. People who identify as being vaccine hesitant can create a construct to cast vaccinators as “the unhealthy other”; these vaccine-hesitant individuals see themselves as an enlightened but persecuted group of healthy and virtuous people [10]. Researchers postulate that those belonging to this group reinforce each other's beliefs through groupthink, confirmation bias, and overconfidence; being surrounded by others with similar beliefs and values lead to further reinforcement that their decision-making was sound [10]. In other words, there is strength in membership and people feel more confident in their decisions, even if they are not based on science, because others agree with them.

People in groups can be more likely to make errors in decision-making because of groupthink. Groupthink occurs when members of a group avoid making a different decision than the commonly expressed views or beliefs of the group [9]. People have a tendency to want to belong and to do so they avoid expressing views that are different from the ones commonly accepted by the larger group. This avoids confrontation, and increases their feeling of belonging. Applying this concept to vaccine hesitancy in the context of a group who express strong anti-vaccination views, membership may depend on conforming to this view or else the individual may feel like they will no longer be accepted as a member of this group. Groups like this can have a tendency to believe they are invincible, help rationalize each other's views, and truly believe they are correct in comparison to non-group members or individuals who do not belong [9].

Sometimes groups can become so powerful that they can take extreme positions, resulting in polarization and further escalating their commitment. Group polarization can occur when a group doubles down on their beliefs creating a situation where people are strongly tied to these views leading to extremism and risky behaviours [9]. From here group polarization can be reinforced and escalation of commitment can occur because the group has a need to confirm their initial position and strengthen it, which increases the group members alignment to that view [9]. For example, governments who enforce vaccine passport systems could unknowingly strengthen group polarization and the position of vaccine hesitant or resistant people. Group members in this situation are more prone to stay aligned with their initial belief and this could cause an escalation of their commitment to vaccine resistance.

Another behavioural driver of vaccine hesitancy is cognitive bias and heuristics. Heuristics are a form of decision-making shortcuts which can be used to simplify complex tasks but may also cause biases or errors [1]. Decision-making models may be used to clarify and understand the process undertaken by an individual when deciding whether or not to accept vaccination. The decision-making models that are applicable to vaccine hesitancy include the bounded rationality model and the intuition or intuitive decision-making model [1]. Both approaches include the use of heuristics, to act as shortcuts in decision-making. They can also help assist when a gap exists between the complexity of the decision or environment in which the individual operates, and cognitive limitations or a lack of information exists.

An example of heuristics is seen in the media coverage of a 73-year-old woman who initially declined the COVID-19 vaccine due to a previous negative experience she had after receiving the influenza vaccine [11]. This situation aligns with the description of intuitive decision-making whereby a person decides based on past experience rather than logic or evaluation of the information, particularly when uncertainty exists [1]. However, bounded rationality decision-making may have also been at play if, upon evaluating the available information, the woman had limitations to her cognitive processes, such as various competing priorities, time pressure, information overload, or other human factors like fatigue that interfered with her ability to thoroughly assess the information to decide on a favourable outcome. In either case, one of the intuitive or bounded approaches led to the implementation of a heuristic. In this person's case, evidence of both availability bias or a version of anchoring and adjustment bias may be seen. She is able to recall a specific example of a negative outcome with a vaccine, in this case, it was her own experience, and then assigns a higher probability of the negative events occurring again from the COVID-19 vaccine due to the ease of recall of her negative experience. This availability heuristic results in the individual failing to consider the difference of the vaccines, influenza versus COVID-19, as well as ignoring the probability of negative effects actually occurring. Instead, she seems to assume that because some side effects occurred before they will occur again, as this example of negative experience is her most available recollection.

A form of anchoring and adjustment may also be seen with this individual, as she seemed to anchor her initial decision to decline vaccination based on the early information available to her and a prior experience of receiving an influenza vaccine. This served as a point of reference. This heuristic caused her to compare new information to her anchor which was eventually adjusted when trusted information became available to her in the form of a webinar held by her physician. This person was able to avoid committing another common bias of escalation of commitment heuristic where an individual continues to rationalize and further commit to a decision that, to others, is clearly unfavourable; however, the individual may not want to admit they were wrong in the initial decision [1]. The escalation of commitment cognitive bias provides some explanation for why we see some individuals remain vaccine hesitant after most of their group or community, in some cases over 70 or 80%, have been vaccinated, and where, despite vaccine mandates, workplaces continue to have some employees that hold out from getting vaccinated.

Recommendations for addressing behavioural drivers of vaccine hesitancy

A number of recommendations can be offered to counter the behavioural drivers of vaccine hesitancy. To deal with the driver of individualism, leaders should attempt to foster and promote collectivist attitudes amongst their population to increase safety in this pandemic and future scenarios [3]. Researchers from this study also postulated that in cases where individualistic tendencies are deeply rooted, the focus would likely need to be on the individual benefits of following public health measures and vaccination instead of collective social responsibility.

Overcoming personal barriers to communication is an important step in addressing multiple behavioural drivers of vaccine hesitancy including individualism and motivation. Personal barriers to communication arise from an individual’s socioeconomic background and lived experiences [1]. These barriers can include an individual’s beliefs, values, prejudices, and frame of reference. These factors can lead an individual or group to block a message which does not fit with their preconceived opinion. To counter this, clear communication and messaging are essential, and the messages must appeal to every individual, and not just those with an altruistic mindset. As discussed in the cited studies, individuals with collectivist beliefs had a higher intention to receive the vaccination, and countries that scored low on the Hofstede individualism measure also tended towards lower COVID-19 mortality rates [3]. For countries like this, messaging should focus on the collective benefits of vaccination and public health measures as these societies are more likely to respond favourably to messaging which focuses on altruism. In contrast, individualistic people were likely to have lower vaccination intention, and individualistic countries also tended to have higher COVID-19 mortality rates [2,3]. This is the group that we must focus on to improve vaccine hesitancy. For these individuals, altruistic messaging alone will not be effective, as individualism is often deep-seated in their cultures. These individuals are less likely to be motivated by societal benefits or how their actions may help others. Instead, the messaging needs to focus on personal benefits from following public health measures and getting vaccinated, such as self-protection and increased freedom of travel; this will likely be more appealing to those who are individualistic and thus, have greater success in increasing vaccination rates. In order to motivate this specific group of people, we must be able to convince them that public health practices and vaccination actually contribute to their overall safety and security, rather than diminishing it.

In addressing the behavioural driver of motivation it is essential to educate vaccine-hesitant individuals about the benefits of population-level immunity and how it impacts their loved ones who are vulnerable to COVID-19 [7]. This recommendation is rooted in content theories of motivation that rely on understanding what drives behaviour and how to arouse, energize, and initiate individual behaviours [1]. By focusing on motivation beyond individual perception of risk and explaining the effect the decision has on family and friends, it can impact extrinsic motivational factors, through considerations of interpersonal relationships, love, and other affiliations. Moreover, concentrating on the greater social benefits of vaccination can create motivation by explaining how an individual's actions can have a substantial impact on the lives of other people. Emphasizing this aspect can instill a sense of meaningfulness in choosing to receive the vaccine, which can improve motivation in those who are vaccine-hesitant due to complacency.

To address the behavioural driver of attitudes in relation to vaccine hesitancy it is important to approach these patients with compassion and understanding and to listen to their concerns. By openly discussing the fears or beliefs that are at the root of this distrust of vaccines, practitioners will be in a better position to defuse patient concerns. Research has shown that vaccine-hesitant parents of children are more accepting of vaccines when their concerns are listened to and addressed [12]. Furthermore, parents who trust physicians as their primary source of information on vaccines are likely to have fewer concerns about vaccination compared to those who rely on sources such as family, friends and the internet [12]. It is reasonable to extend this approach to individuals who are hesitant of receiving the COVID-19 vaccine as the doctor-patient relationship often entails some level of trust and open communication. Although physicians may not be the primary vaccinators in public health settings, they can still be a trusted source of evidence-based information and health dialogue for many patients.

Perceptions are another key behavioural driver of vaccine hesitancy and healthcare providers play a critical role in addressing this driver due to their strong influence on public perceptions. More specifically, research shows 86% of Ontarians view physicians as a trusted source of public health advice related to COVID-19 vaccination [8]. Individualized and tailored approaches to address factors that influence a person’s decision to receive the vaccine should be implemented, with conversations starting from a place of empathy and respectful dialogue that avoids polarizing labels such as "anti-vaxxers" and "conspiracy theorists". Framing immunization in terms of positive gains while addressing concerns about risks can help to build trust, confidence, and transparency. Healthcare providers should address any misinformation by sharing evidence-based health practice with patients and making a point to proactively explain side effects. It is important for individuals to understand the risks and benefits through their own engagement with credible sources of information to address their personal perceptions and attitudes regarding vaccine safety and efficacy.

To combat the driver of groupthink healthcare providers should have non-judgmental conversations with their patients about vaccination to foster a different frame of reference from their group beliefs, provide a safe space, and provide information to challenge their decision-making process. If the group is progressing to showing signs of group polarization, one potential way to combat this challenge is to encourage people in decision-making positions to be inclusive and express a more diverse view rather than enforcing policy that will penalize members for non-conformity [9].

To combat the behavioural drivers of heuristics and cognitive biases it is essential to gain a better understanding of an individual’s decision-making process. This insight can then be applied to generate potential interventions to address vaccine hesitancy. Knowing these decision-making models and heuristics unconsciously being used allows us to tailor interventions specific to the individual. The aforementioned example of the 73-year-old woman shows how trusted information, delivered in a non-judgmental, compassionate, and person-centered manner, can be effective against vaccine hesitancy. Consideration should be given to implementing motivational interviewing techniques such as showing empathy, using active listening and summarizing statements, avoiding making assumptions, using semi-directive or presumptive statements and open-ended questions [12]. If the person is able to see and understand a discrepancy exists between what their own goals are and where they’re currently positioned, it may become clear that a position or strategy change is needed to break free from the heuristic at play. These techniques ought to be considered in working with risk-averse individuals who are fearful of negative side effects of vaccination.

In developing a deeper understanding of these behavioural drivers, it is evident that vaccine hesitancy is a complex and multifaceted issue. As such, the recommendations that can be implemented by healthcare providers to increase vaccine acceptance must be specific and tailored to the underlying drivers of vaccine hesitancy. For example, utilizing extrinsic motivation and emphasizing societal benefits can be effective in combating vaccine hesitancy in those who are complacent; whereas this may be ineffective in addressing individualism, in which case messaging that focuses on personal benefits of vaccination should be utilized. One overarching recommendation is for clear communication; this involves overcoming the personal barriers to communication in a supportive and compassionate manner, to openly discuss and address the perceptions, attitudes, and beliefs individuals have developed that influenced their decision not to receive the COVID-19 vaccine.

Effective population-level messaging should be tailored to appeal to individuals with either collectivist or individualistic beliefs. People in decision-making positions should make efforts to be inclusive and express more diverse views to address groupthink and polarization. Moving forward, it is recommended that leaders should attempt to foster and promote collectivist attitudes among their population, which can help in future pandemics where the challenges of vaccine hesitancy may persist. Ultimately, by understanding the drivers behind vaccine hesitancy from an organizational behaviour perspective, these recommendations can be utilized to support individual uptake of vaccines in various settings.
